# Comparative Study of Pd-Mayenite Catalysts Prepared via Aerogel Approaches

**DOI:** 10.3390/gels8120809

**Published:** 2022-12-10

**Authors:** Ekaterina V. Ilyina, Alexander F. Bedilo, Grigory B. Veselov, Yuri Y. Gerus, Ekaterina I. Shuvarakova, Vladimir O. Stoyanovskii, Aleksey A. Vedyagin

**Affiliations:** 1 Boreskov Institute of Catalysis SB RAS, Novosibirsk 630090, Russia; 2 Department of Natural Sciences, Novosibirsk State University, Novosibirsk 630090, Russia

**Keywords:** aerogel technique, mayenite, palladium-containing catalysts, CO oxidation, UV–Vis spectroscopy, temperature-programmed reduction

## Abstract

Pd-containing catalysts based on highly dispersed aerogel-derived mayenite were prepared via two approaches. The Pd@C12A7 sample was obtained through the addition of Pd nitrate solution to a fresh Ca(OH)_2_-Al(OH)_3_ gel. Pd/C12A7 was synthesized through conventional wet impregnation of the aerogel-derived mayenite. The evolution of the textural characteristics of the support (C12A7) depending on the calcination temperature was investigated. Pd-containing samples were explored using transmission electron microscopy and spin probe EPR spectroscopy. Using the latter method, the presence of active oxygen species capable of producing nitroxyl radicals from diphenylamine was observed. The activity of these species and the reproducibility of their redox behavior were studied in three cycles of temperature-programmed reduction in both hydrogen and CO atmospheres. A prompt thermal aging technique was used to access and compare the activity of the samples towards CO oxidation. The state of Pd species before and after the aging procedure was studied via UV–Vis spectroscopy. It was found that the dispersion of PdO was higher in the case of the Pd/C12A7 catalysts compared to the Pd@C12A7 sample. This is why the Pd/C12A7 catalyst demonstrated higher activity in CO oxidation and better reducibility in TPR cycles.

## 1. Introduction

Recently, there has been a growing interest in calcium aluminate systems with a mayenite structure. The stoichiometric formula of mayenite is 12CaO·7Al_2_O_3_, usually denoted C12A7. The properties of mayenite are determined by its unique structure. Its cubic unit cell can be described as [Ca_24_Al_28_O_64_]^4+^·2O^2−^, where the first part serves as a framework containing 12 cages and O^2−^ are extraframework ions occupying 2 of 12 framework cages [1]. C12A7 synthesized in an oxidative medium, depending on the conditions, may contain, apart from O^2−^, such anions as OH^−^ and active forms of oxygen O^−^, O_2_^2−^, and O_2_^−^. These ions can be substituted for F^−^, Cl^−^, S^2−^, H^−^_,_ and NO_3_^−^ ions or even for free electrons [2,3,4]. Therefore, the properties of C12A7 depend significantly on the type of encaged anion.

There are many reports in the literature dedicated to the application of mayenite as a catalyst support. For instance, a Pd/mayenite catalyst was shown to catalyze the reduction of benzaldehyde to benzyl alcohol with a high selectivity. In order to prepare the catalyst, Pd was deposited on the surface of mayenite through its reduction from PdCl_2_ solution in the presence of NaBH_4_. The catalyst synthesized in this manner demonstrated better conversion and selectivity than the commercial Pd/C catalyst [5]. Yang et al. studied calcium aluminate-based catalysts for methane partial oxidation with different metals used as an active component. The dispersion of nickel on C12A7 was the highest compared to calcium aluminates with other stoichiometries. With the presence of encaged oxygen in mayenite, this provides efficient performance in C12A7-based systems. Thus, the highest activity and stability were observed for Pt/C12A7 and Ni/C12A7 catalysts, even with low temperature, low metal loading, and high space velocity [6]. The presence of sub-nanometer cavities on the surface of mayenite allows the stabilization of single Pt atoms [7]. During the impregnation of mayenite with a [PtCl_4_]^2−^ solution, [PtCl_4_]^2−^ ions anchor to the surface and, subsequently, their reduction leads to the formation of Pt atoms inside these cavities. Such catalysts exhibited high activity and selectivity in the hydrogenation of nitroarenes.

Among the most prominent mayenite-based systems, Ni/C12A7 catalysts should be mentioned. They are primarily used for steam and dry reforming of methane and tar [8,9,10,11,12,13,14]. Their high efficiency is a result of their high resistance against coke formation and sulfur poisoning. Reactive oxygen species present in mayenite play a key role in preventing these processes [9]. The preparation of nickel-based catalysts is usually carried out with a wet impregnation technique. The type of the precursor salt used affects the metal–support interaction significantly. Thus, catalysts prepared using nickel nitrate were found to be more active than those prepared using nickel acetate [10]. In work by Bracciale et al. [11], a Ni/C12A7 catalyst prepared using the impregnation method was compared to that prepared using a “one-step” route when nickel nitrate was introduced directly into a mixture of hydroxide precursors. The sample prepared with this route was less active but also less prone to coke formation and, therefore, more stable. Another way to reduce the coke formation is doping of the catalyst with iron [15]. Surprisingly, doping with CeO_2_ diminishes the activity of Ni/mayenite in tar reforming but slightly increases its activity when sulfur-containing compounds are present in the reaction mixture [14].

As stated above, various oxygen species, such as OH^−^, O_2_^2−^, O^2−^, and O_2_^−^, can be stored in mayenite cages. In the context of the practical use of mayenite and catalysts based on it, it is interesting to study the reactivity of these forms with respect to various reducing agents, as well as the conditions for their regeneration. The presence of reactive oxygen species determines the oxygen storage capacity (OSC), which is important in various catalytic processes. In particular, OSC is one of the key characteristics of three-way catalysts, in which the role of the oxygen storage component is usually played by CeO_2_ and systems based on it, such as Ce_1−x_Zr_x_O_2_ [16,17,18]. For the characterization of OSC, among other properties, the temperature-programmed reduction (TPR) method can be used, and the use of various reducing agents (H_2_ and CO) can provide more complete information about the reactivity of reactive oxygen species. As reported by Intiso et al. [19], a correlation was found between the hydrogen uptake in TPR and the activity of mayenite in the total oxidation of trichloroethylene. Compete oxidation can be achieved due to the high reactivity of the oxygen species present in mayenite. On the other hand, the poisoning of the catalyst with chlorine occurs. Doping with Fe has been found to facilitate the reduction of oxygen species in mayenite, which was linked to better catalytic performance [20]. However, other researchers failed to detect a significant effect from Ni and Fe doping on the rate of oxygen surface exchange in mayenite [21]. Yang et al. investigated the desorption of oxygen species and their regeneration in mayenite prepared through a ceramic route in detail [22]. Desorption proceeds in three steps: diffusion of oxygen species through the bulk of mayenite; their decomposition on the surface; and release of molecular oxygen into the gas phase. The first step was determined to be the rate-limiting one, implying that an increase in the dispersity of mayenite would facilitate desorption of the oxygen species.

Obviously, to attain maximum efficiency as a support, it is necessary to achieve the optimal textural characteristics in the mayenite. Conventional methods for the preparation of mayenite involve high temperature solid-phase synthesis, resulting in the production of non-porous materials [22]. The development of low-temperature preparation methods that retain the good textural characteristics of mayenite is very important, especially for catalytic applications. The aerogel approach to preparing finely dispersed materials is based on a sol-gel technology, which involves hydrolysis of an organometallic precursor with the formation of a gel of the corresponding hydroxide. This method is of considerable interest because it avoids the collapse of the porous structure of the synthesized sample by removing the solvent under supercritical conditions in an autoclave [23,24,25]. Recently, we synthesized aerogels of calcium aluminate with a mayenite stoichiometry for the time first [26]. The specific surface area of such samples reached 170 m^2^/g after calcination at 500 °C. The obtained small mayenite crystallites were mainly X-ray-amorphous. By varying the Ca:Al molar ratio, samples with different phase compositions were obtained [27]. The sample with the stoichiometry of mayenite (Ca:Al = 6:7) was found to be the most promising one for application as a catalyst support.

In the present manuscript, the preparation of Pd-containing C12A7-based catalysts and an investigation of their catalytic properties are reported. The samples were prepared via two different synthetic approaches. First, the addition of palladium salt to a freshly prepared Ca(OH)_2_-Al(OH)_3_ gel was used. The advantages of this approach have been previously shown in relation to VO_x_/MgO aerogels [28]. Second, wet impregnation of the aerogel-derived C12A7 support with a solution of palladium salt was utilized. The samples were characterized with such methods as low-temperature nitrogen adsorption, X-ray diffraction (XRD), electron paramagnetic resonance spectroscopy (EPR) using spin probes, and transmission electron microscopy (TEM). The oxygen storage capacity of the catalysts was characterized via temperature-programmed reduction (TPR) in a hydrogen or CO atmosphere. Catalytic performance in CO oxidation was explored in a prompt thermal aging (PTA) regime.

## 2. Results and Discussion

### 2.1. Textural Characteristics of the Samples

**Figure 1:**
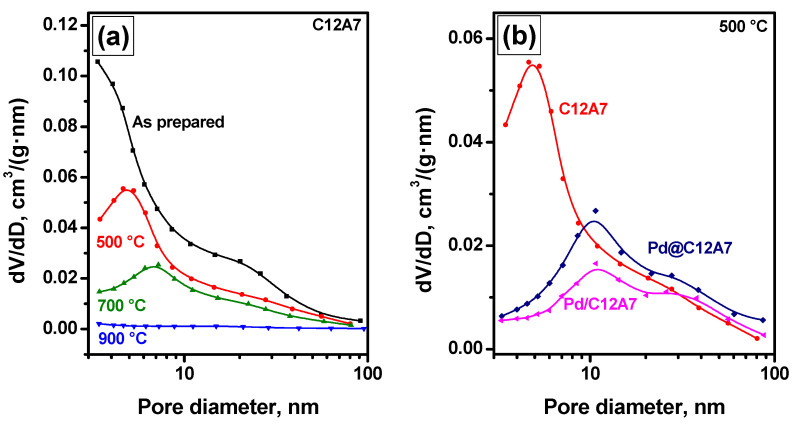
Pore size distributions for pure C12A7 calcined at different temperatures (**a**) and its comparison with Pd-containing C12A7 samples calcined at 500 °C (**b**).

It is known that prolonged operation of any catalyst at elevated temperatures can affect its textural characteristics significantly. Using mayenite as a support, it was interesting to monitor the evolution of these characteristics for pure and Pd-containing C12A7 depending on the calcination temperature. Figure 1a shows the pore size distributions for pure, freshly prepared C12A7 calcined at temperatures in the range of 500 to 900 °C. As can be seen, an increase in the calcination temperature decreased the pore volume and shifted the maximum of the pore size distribution towards larger sizes. The data presented in Table 1 show that the specific surface area (SSA) diminished as well. It is worth noting that, even after calcination at 700 °C, the SSA value was still as high as ~150 m^2^/g, which noticeably exceeds those of mayenite samples derived with the other methods [29]. Only the calcination at 900 °C resulted in significant sintering accompanied by a sharp drop in SSA and pore volume.

Table 1 also shows the textural characteristics of Pd/C12A7 and Pd@C12A7 samples calcined at 500 °C. As can be seen, the introduction of palladium, regardless of the preparation route, diminished the SSA value to 200 m^2^/g and below. The pore volumes of both palladium-containing samples were slightly higher than that of the C12A7 sample. In terms of the pore size distribution (Figure 1b), the introduction of palladium decreased the volume of smaller pores (below 9 nm) and increased the volume of bigger ones (30 nm and above). It is worth noting that a decrease in SSA due to addition of salts at the hydrolysis stage during sol-gel synthesis was observed recently for MgO-based systems [30]. Since the textural characteristics of the Pd/C12A7 and Pd@C12A7 samples were quite similar, no noticeable difference in the catalytic performances of the samples was expected.

### 2.2. TEM Study

**Figure 2:**
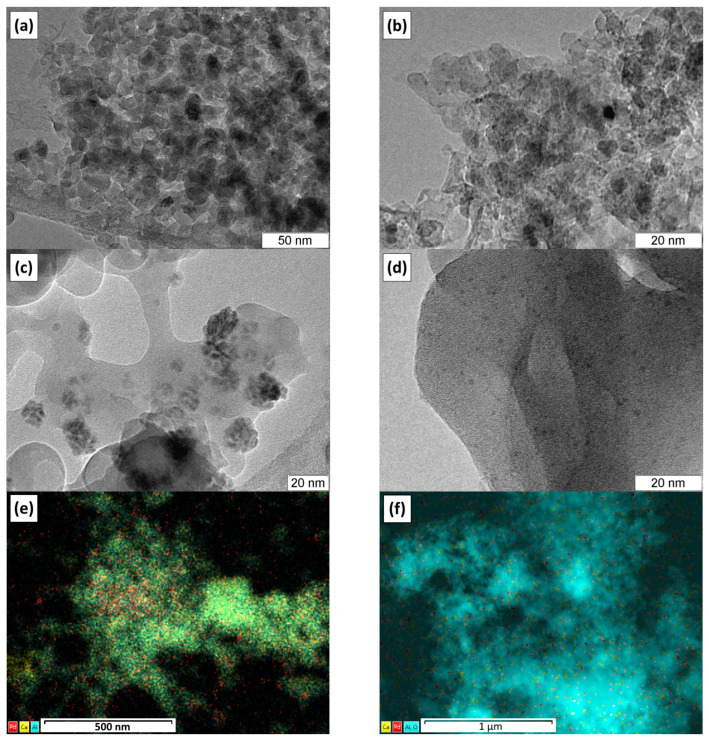
HR TEM images after minimal exposure to an electron beam (**a**,**b**) and after exposure to the electron beam (**c**,**d**), as well as elemental mapping (**e**,**f**), for the Pd-containing C12A7 samples: Pd@C12A7 (**a**,**c**,**e**); Pd/C12A7 (**b**,**d**,**f**).

Both the Pd@C12A7 and Pd/C12A7 catalysts were studied using transmission electron microscopy (Figure 2). The morphology of the samples was represented by platelets of ~5 nm in size. Despite the Pd species being hardly distinguishable in the microscopic images, their presence was confirmed by the EDX elemental analysis. It can be assumed that palladium was present in the form of very small PdO clusters. Elemental mapping of these samples revealed that palladium was evenly distributed within the bulk of the Pd@C12A7 (Figure 2e). Calcium and aluminum were fully mixed, appearing as a single green color. The palladium content was estimated to be 1.17 ± 0.12 wt%, and this value was reproduced with high accuracy for a number of TEM images. For the impregnated sample Pd/C12A7 (Figure 2f), palladium was localized non-uniformly, and its content varied in the range from 1 to 4 wt% in different areas of the sample. Calcium aluminate was partially fractionized, and aluminum and calcium were observed separately as yellow (calcium) and blue (aluminum) areas.

One interesting feature of these samples should be mentioned. On the one hand, this feature makes it difficult to study them using transmission microscopy. On the other hand, it can help in their further characterization. Under the action of an electron beam, the particles of the mayenite support sinter and increase in size. For the Pd@C12A7 sample, palladium appeared on the surface in the form of lace-like large prints, reaching 20 nm in size (Figure 2c). In the case of the Pd/C12A7 sample, particles ~1 nm in size were observed (Figure 2d). Therefore, depending on the method used for the introduction of palladium, the samples differed both in morphology and in the stabilization of palladium under extreme conditions.

### 2.3. EPR Spin Probe Characterization of the Samples

**Figure 3:**
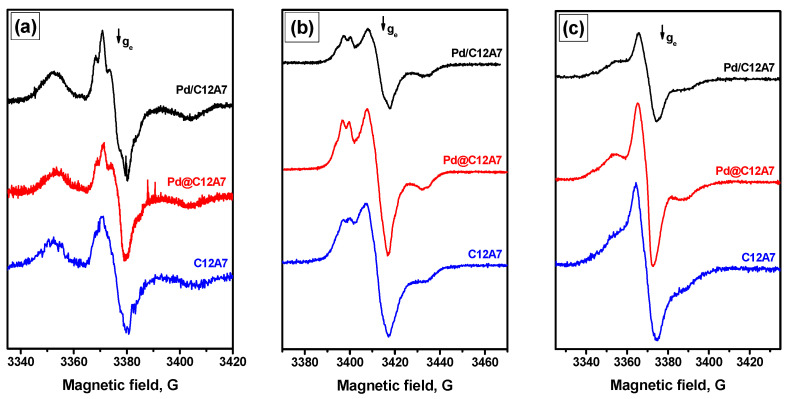
EPR spectra of Pd/C12A7 and Pd@C12A7 samples after adsorption of different spin probes: (**a**) TNB; (**b**) PTZ; (**c**) DPA.

Surface active sites were studied with EPR using different spin probes. The EPR spectra observed after adsorption of 1,3,5-trinitrobenzene (TNB) on the Pd-containing mayenite samples are shown in Figure 3a. As can be seen, the spectra for Pd-containing samples were similar to the spectrum for pure C12A7, which was described in detail recently [27]. In general, all these spectra are reminiscent of the spectrum observed for alumina after TNB adsorption attributed to an ion pair of a TNB radical anion and aluminum cation [31]. Note that the *A*_zz_ parameter has a somewhat smaller value of 27 G (vs. ~30 G for Al_2_O_3_). This observation indicates that electron-donor sites were present on the surface of Pd-containing mayenite samples. Similar spectra are also typical for nanocrystalline mayenites prepared through the reaction of CaO with Al hydroxide in water [32]. The concentration of these sites was estimated to be of ~0.5·10^18^ g^−1^ for both the samples with palladium. This value was slightly lower than that for pure C12A7 (Table 2). It is important to mention that the heating of the samples did not lead to significant changes in the concentration of the observed radical anions. Apparently, Pd nanoparticles compete with TNB for electrons, preventing the formation of TNB radical anions in the complex process involving the solvent molecules that takes place during the heat treatment. It should also be noted that an even lower concentration of TNB radical anions was observed over Pd@C12A7 compared to the impregnated sample (Pd/C12A7).

**Figure 4:**
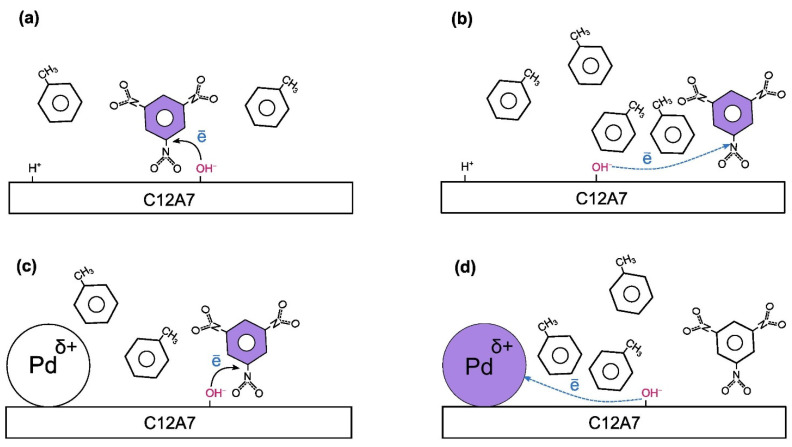
Scheme of the formation of TNB radical anions after interaction with surface donor-sites. The final electron acceptor is shown in purple. (**a**) Fast formation of a TNB radical anion after adsorption over C12A7. (**b**) Slow formation of a TNB radical anion over C12A7 after heating. (**c**) Fast formation of a TNB radical anion after adsorption over Pd-C12A7. (**d**) Slow electron transfer to Pd nanoparticle instead of TNB over Pd-C12A7.

It has been suggested that electron-donor sites could be related to surface OH^−^ groups located at some distance from the positive counter ions, such as H+ [33]. A scheme of the formation of TNB radical anions after interaction with surface donor sites is shown in Figure 4. The TNB molecule that captures an electron to form a radical anion immediately after adsorption would probably be located next to the surface OH^−^ group acting as an electron-donor site (Figure 4a). Those that are revealed as radical anions only after heating would be located at some distance away, and this process would require some intermediacy by solvent molecules (Figure 4b).

Pd clusters and nanoparticles stabilized on the surface of oxide supports are known to have partial positive charge. As a result, they can act as a competitor to the TNB molecule in capturing electrons from electron-donor sites. Nevertheless, if the electron transfer from an electron-donor site to a TNB molecule is fast, Pd nanoparticles can hardly intervene in this process to a significant degree, as their concentration is quite low (Figure 4c). As a result, the addition of Pd had little effect on the concentration of sites observed immediately after TNB adsorption (Table 2). Moreover, when the electron transfer is a slow, complex reaction involving solvent molecules, the Pd nanoparticle might end up being a better electron acceptor than the TNB (Figure 4d) and reduce the number of TNB radical anions observed after heating at 80 °C.

In order to characterize weak electron-acceptor sites, probe molecules with low ionization potential should be used. Phenothiazine (PTZ), having a potential of ~6.8 eV, is suitable for this purpose. The EPR spectra recorded after adsorption of PTZ can be assigned to phenothiazine radical cations (Figure 3b). The *A*_zz_ parameter for these spectra is 18 G. The calculated concentration of weak electron-acceptor sites was ca. 1.5 times higher on the Pd@C12A7 sample compared to the Pd/C12A7 sample (1.34·10^18^ g^−1^ vs. 0.85·10^18^ g^−1^, Table 2). In general, the addition of palladium noticeably decreases the concentration of radical cations observed after PTZ adsorption. The heat treatment of the Pd-containing samples at 80 °C did not increase the concentration of the radical cations. It can be assumed that the Pd ions present on the surface provide for an additional charge transfer pathway, leading to a substantial decrease in the concentration of radical species.

Figure 3c shows the EPR spectra observed after DPA adsorption on the samples under study. In this case, the *A*_zz_ parameter is 16 G. Such spectra were described recently [26,27]. It is worth noting that the triplet in these spectra, which is related to surface diphenyl nitroxide radicals [26], testifies to the presence of active O^−^ or peroxyl radicals on the surface of the Pd-containing samples. Again, the concentration of radical species for the Pd@C12A7 sample exceeded that for the Pd/C12A7 sample by a factor of ca. 1.4 (Table 2).

The data obtained using the EPR spin probe technique suggest that the introduction of Pd diminished the concentrations of all radical species observed after adsorption of probe molecules. This observation is somewhat different from observations of the behavior of Pd/Al_2_O_3_ catalysts reported previously [34]. The introduction of Pd to Al_2_O_3_ is known to increase the concentration of electron-donor sites but does not generally affect electron-acceptor cites. Although amorphous γ-Al_2_O_3_ was likely to be present in the samples, it did not play a large role in Pd stabilization.

The phase composition of aerogel-prepared mayenite has been studied and reported previously [26,27]. It should be mentioned that, when a stoichiometric amount of water was used, the mayenite phase was amorphous to X-rays. The changes in phase composition related to the introduction of Pd seem to be insignificant. The presence of the mayenite phase in these samples was confirmed by the observation of characteristic active oxygen species using the spin probe EPR technique.

### 2.4. Temperature-Programmed Reduction Experiments

In this work, the reactivity of oxygen species was also studied by means of temperature-programmed reduction (TPR) in two atmospheres, H_2_ and CO. The samples were studied in three consecutive reduction/oxidation cycles. Each TPR stage was followed by regeneration with air at 500 °C. This allowed the estimation of the reproducibility of the redox behavior of the samples.

**Figure 5:**
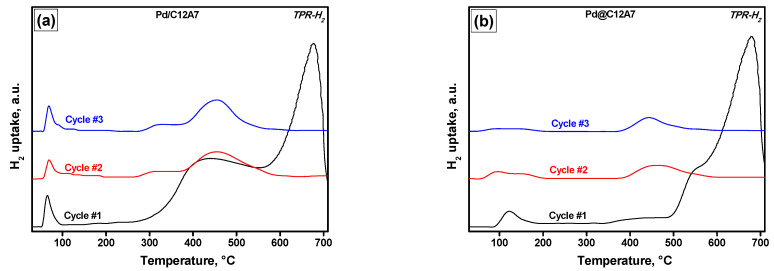

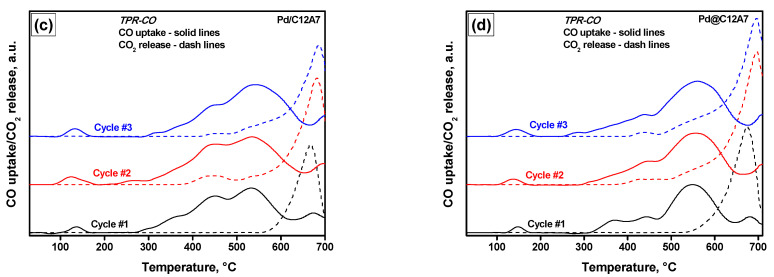
TPR-H_2_ (**a**,**b**) and TPR-CO (**c**,**d**) profiles for the Pd-containing samples: Pd/C12A7 (**a**,**c**); Pd@C12A7 (**b**,**d**).

The TPR-H_2_ profiles of the Pd/C12A7 and Pd@C12A7 samples are shown in Figure 5a,b. One low-temperature peak can be clearly seen in each profile. For the Pd/C12A7 sample, this peak appeared at a lower temperature (50–100 °C) compared to the Pd@C12A7 sample (100–150 °C). Such a difference in the reduction behavior may be connected to various reasons, such as dispersion of PdO species, their accessibility to the reducing agent, and the strength of their interaction with the support. These results correlate well with the TEM data.

For both samples, the hydrogen uptake at temperatures below 200 °C was close to the theoretical value of 93 µmol/g (Table 3), thus indicating that the palladium was mostly in the oxidized state. The hydrogen consumption at higher temperatures was assigned to the reduction of the various oxygen species contained in mayenite. For the impregnated sample, the uptake value appeared to be noticeably higher, which was mainly due to the more intensive uptake in the temperature range of 400 to 500 °C. At the same time, for both samples, there was a peak of about the same intensity, with a maximum at ~650 °C. According to the literature, hydrogen uptake by mayenite occurs mainly within the temperature range of 500 to 650 °C [19]. This may be associated with the reduction of various extraframework oxygen species, as well as the inclusion of hydrogen in these cages in the form of H^−^ ions.

For the Pd/C12A7 sample, the shape and the intensity of the PdO peak in redox cycles remained substantially unchanged. At the same time, for the Pd@C12A7 sample, this peak widened from cycle to cycle with a noticeable loss in intensity. The uptake peak associated with the reduction of extraframework mayenite ions was shifted in the second and third cycles to the temperature range of 300 to 550 °C, with a pronounced maximum at ~450 °C. In general, between these two samples, Pd/C12A7 exhibited better reproducibility in redox cycles, along with higher uptake values. At the same time, for both Pd-containing samples, these characteristics exceeded those of the C12A7 samples prepared from a hydroxide precursor via a non-aerogel approach [35]. All of this may be due to the advantages of the aerogel technique as expressed in the tailored surface area of the materials and the better accessibility of oxygen species for reagents.

The TPR-CO profiles corresponding to the Pd/C12A7 and Pd@C12A7 samples are shown in Figure 5c,d. It is important to note that the TPR-CO experiments were performed immediately after the TPR-H_2_ tests. Therefore, no noticeable differences in the consecutive redox cycles in the CO atmosphere were expected. As in the case of TPR-H_2_, two temperature intervals can be distinguished. The first one lies below 200 °C and corresponds to the reduction of palladium oxide species. The second one, assigned to the reduction of ions in the mayenite lattice, appears at temperatures above 300 °C. However, there are several peaks in the profiles that cannot be unambiguously assigned to the reduction of certain oxygen species. The CO uptake values were higher in the case of the Pd/C12A7 sample in all three cycles. It is also worth noting that the CO uptake values were markedly superior in relation to the H_2_ uptake values. This may indicate a greater affinity of reactive oxygen species with respect to CO. However, the true reason for this phenomenon remains unclear. For example, the Boudouard reaction (2CO ↔ CO_2_ + C) proceeds over nickel, cobalt, iron [36], and some other metals but not over Pd. On the other hand, an interesting feature of these samples is the presence of CaO in them. For both samples, CO_2_ was not released in the first TPR-CO cycle at temperatures below 550 °C, which is obviously connected with its strong chemisorption on calcium oxide and the formation of surface carbonates. Starting from the second cycle, the CO_2_ desorption temperature shifted to ~450 °C. Despite the higher CO uptake values of the Pd/C12A7 sample, a larger amount of released CO_2_ was recorded for Pd@C12A7. It is worth noting that, in both cases, the CO_2_ molecules did not desorb completely. This was predictable, since the decomposition of CaCO_3_ occurs at temperatures above 700 °C [37]. The Pd/C12A7 sample was characterized by what was probably the presence of a larger number of strong basic sites capable of strong CO_2_ adsorption. As already mentioned, this sample was characterized by a higher concentration of electron-donor sites, as determined by the EPR spin probe technique after the TNB adsorption.

Summarizing the TPR data, the following statements can be made. In the case of the Pd/C12A7 sample, the highly dispersed PdO species were distributed over the outer surface of the mayetine crystallites, which resulted in simplification of their reduction, as well as more reproducible behavior in the TPR cycles. The presence of Pd on the surface could also facilitate the reduction of ions encaged in the mayenite lattice. Thus, more efficient hydrogen spillover from Pd particles to the support and more efficient CO adsorption on these particles can take place in the cases of TPR-H_2_ and TPR-CO, respectively.

### 2.5. Catalytic Performance of the Samples

In general, catalytic oxidation of CO over palladium proceeds via the formation of adsorbed CO and oxygen species followed by interaction between them [38]. In our case, oxygen-rich mayenite could provide an increased amount of surface oxygen species, thus improving the catalytic performance.

**Figure 6:**
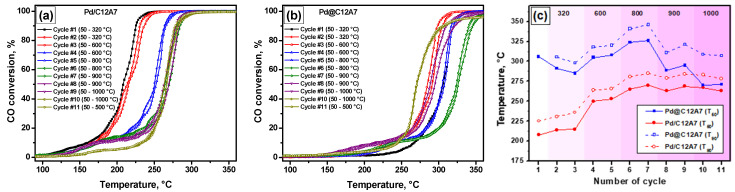
Light-off curves in the PTA mode for the samples: (**a**) Pd/C12A7; (**b**) Pd@C12A7. Comparison of the corresponding T_50_ and T_90_ values for these samples (**c**).

In the present work, the catalytic performance of the samples in CO oxidation was explored in a prompt thermal aging (PTA) mode. This procedure involves testing a sample in several heating–cooling cycles. The temperature at the final heating point is increased in each second cycle, thus reaching 320, 600, 800, 900, and 1000 °C in the second, fourth, sixth, eighth, and tenth cycles, accordingly. The observable changes in the catalytic activity reflect the evolution of the active sites of the catalysts caused by the thermal treatment at different temperatures. The results of the catalytic experiments are summarized in Figure 6.

In the case of the impregnated sample Pd/C12A7 (Figure 6a), the CO conversion curve corresponding to the initial state of the sample (first cycle) lies in a low-temperature region. After such contact with the reaction mixture at 320 °C, the light-off curve shifts to higher temperatures, indicating a decrease in the activity. The next deactivation step is seen after the aging at 600 °C. Then, in the range of 800 to 1000 °C, the sample exhibits more or less stable behavior. It should be emphasized that the specific surface area of the support dropped during such a treatment from ~250 to ~20 m^2^/g (Table 1), with no noticeable effect on the activity. All the light-off curves have a shoulder in the low-temperature region, which is consistent with the TPR-CO data revealing a CO uptake at 100–150 °C.

The catalytic performance of the Pd@C12A7 sample in the PTA mode differed significantly from that of the Pd/C12A7 sample (Figure 6b). In order to compare the samples more clearly, the temperatures of the 50% conversion (T_50_) in consecutive cycles were plotted (Figure 6c). As can be seen, the Pd@C12A7 sample showed significantly worse initial activity compared to the impregnated analog. The difference in the T_50_ values was about 100 °C. The initial activity of the samples agreed well with TEM data, demonstrating better dispersion of the Pd species in the case of the impregnated sample. According to the EPR characterization, this sample possessed a higher concentration of electron-donor sites. As we have reported previously [34,39], the concentration of such sites correlates with the activity of Pd/Al_2_O_3_ and Pd/ZrO_2_ catalysts in CO oxidation. An increase in the activity of the Pd@C12A7 sample was observed in the second and third cycles. Then, after aging at 600 and 800 °C, a two-step deactivation was evident. The high-temperature treatment (900–1000 °C, eighth–eleventh cycles) reactivated the sample to the same level as shown by the Pd/C12A7 sample. It can be assumed that the loss in the specific surface area of mayenite, along with the changes in its pore structure, made the palladium species more accessible for the reactants. At the same time, the dispersion of these species increased, which follows from the comparison of the 90% conversion (T_90_) values. The phenomenon of palladium redispersion at elevated temperatures is well-known from the literature [40,41,42,43,44]. Pd/C12A7 aged at 1000 °C showed a T_90_ value of 278 °C vs. 307 °C exhibited by the Pd@C12A7 catalyst. Therefore, the Pd/C12A7 sample prepared through the impregnation of the aerogel-prepared mayenite was more prominent in oxidation processes compared to the aerogel-prepared Pd@C12A7 sample.

It should be mentioned that, in terms of catalytic performance, the Pd/C12A7 sample was comparable or even superior with respect to alumina-supported catalysts containing precious metals. Such a comparison with mono-, bi-, and tri-metallic systems tested under similar conditions is given in Table 4.

### 2.6. Diffuse Reflectance UV–Vis Spectroscopy and XRD Data

The state of palladium in the as-prepared samples and those tested in the PTA-mode was characterized using diffuse reflectance UV–Vis spectroscopy. In order to interpret the recorded spectra correctly, the aerogel-derived C12A7 support was investigated first.

Extraframework anions in mayenite form an edge or absorption bands below the fundamental edge associated with the transition from the top of the valence band to the bottom of the framework conduction band (VB → FCB, *E*_g_~6.8 eV). The adsorption edge of mayenite depends on the type of extraframework ions and can range from 4 to ~6 eV. It is shifted towards lower energies with the incorporation of ions in the following order: OH^−^ > O^2−^ > oxygen radical species.

**Figure 7:**
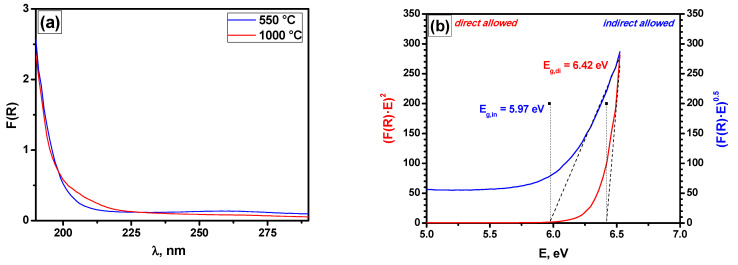
Diffuse reflectance UV–Vis spectra of the C12A7 sample calcined at 550 and 1000 °C (**a**) and dependencies of (*F(R)·E*)^2^ and (*F(R)·E*)^0.5^ on the photon energy *E* characterizing the band-gap *E*_g_ values for direct and indirect allowed transitions for C12A7 (**b**).

Figure 7 presents the UV–Vis spectra for the C12A7 sample after calcination at 500 °C and 1000 °C and the dependencies of (*F*(*R*)·*E*)^2^ and (*F*(*R*)·*E*)^0.5^ on the photon energy *E* characterizing the band-gap (*E*_g_) values for both direct and indirect allowed transitions determined via the Tauc method. The observed value of *E*_g_ ~6.42 eV was lower than the band-gap values of the mayenite framework (~6.8 eV) and of γ-Al_2_O_3_ (~7.6 eV [47]), despite the significant amount of Al_2_O_3_ in the C12A7 sample. Note that the indirect band gap of CaO is 6.4 eV [48]. On the other hand, the proximity to the edge of the measured wavelength range of 190 nm may have possibly lad to errors in the measurements of the *E*_g,dir_ values.

**Figure 8:**
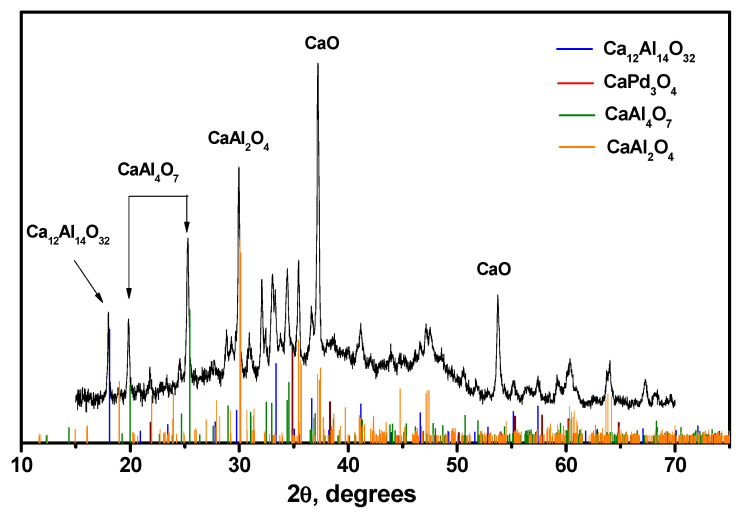
XRD pattern of the Pd/C12A7 sample after catalytic tests in the PTA mode.

The calcination of the C12A7 support at 1000 °C (Figure 7a) did not lead to significant changes. The values *E*_g,dir_ = 6.38 eV and *E*_g,indir_ = 6.05 eV indicate that the formation of the C12A7 phase occurred. This correlates well with the XRD data presented in Figure 8 for the Pd/C12A7 sample after catalytic tests. A number of phases can be observed for this Pd/C12A7 sample: CaO, Ca_2_Al_2_O_4_, CaAl_4_O_7_, and Ca_12_Al_14_O_32_. The corresponding crystallite sizes calculated via the Sherrer equation were 35 nm, 40 nm, 33 nm, and 64 nm. Moreover, no traces of known Pd-containing phases (PdO, CaPd_3_O_4_) were observed, which was presumably connected with the low content of Pd in the studied samples.

**Figure 9:**
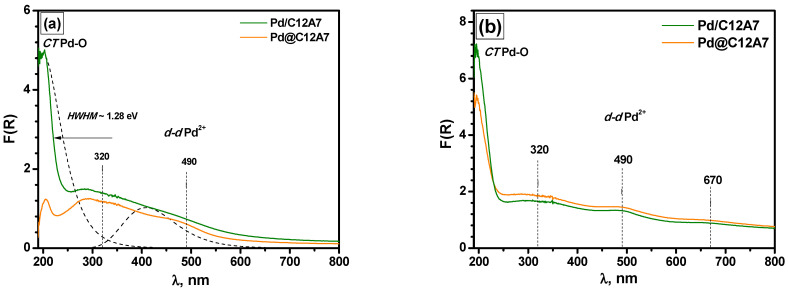
Diffuse reflectance UV–Vis spectra for Pd/C12A7 and Pd@C12A7: (**a**) as-prepared samples; (**b**) samples after catalytic tests in the PTA mode. Dashed lines show the characteristic charge transfer (*CT*) bands and *d-d* transition bands for Pd^2+^ in square-planar coordination, which is typical for PdO clusters.

Figure 9a presents diffuse reflectance UV–Vis spectra for the as-prepared Pd/C12A7 and Pd@C12A7 samples. The Pd-O charge transfer bands at 250 nm and the *d-d* transition bands of Pd^2+^ at 450 nm [49,50] in square-planar coordination for PdO clusters on the Al_2_O_3_ support with a characteristic band-gap value of ~2.35 eV [51] are shown with dashed lines. The band-gap energy value for PdO bulk particles is significantly lower and, according to optical transmittance measurements, equal to ~0.8 eV [52], while electric conductivity measurements have indicated an energy gap value of ~1.5 eV [53].

In CaPd_3_O_4_, each palladium ion is surrounded by four oxygen ions in square-planar geometry: Pd-O = 2.03 Å [54]. This arrangement is very similar to that found for PdO, where Pd ions are located at the centers of rectangles with oxygen ions at each corner and with a Pd-O distance of 2.02 Å [55]. Pristine sintered pellets of CaPd_3_O_4_ display semiconducting behavior, with a room-temperature resistivity of 0.1 Ω cm and a narrow band gap *E*_g_~0.25 eV obtained from transport measurements [56]. There are no reports in the literature concerning band-gap values for nanometer-sized CaPd_3_O_4_ particles or their absorption spectra in the UV–Vis range.

For both as-prepared samples, two absorption bands were observed in the *d-d* transition region at 320 and 490 nm, as well as a charge transfer band at ~200 nm (Figure 9a). However, compared to PdO oxide clusters (HWHM~2.28 eV), this band had a significantly lower HWHM value of 1.28 eV. The difference between the Pd/C12A7 and Pd@C12A7 samples was the lower intensity of the charge transfer band of the latter, which was associated with a partial shielding of Pd-containing particles by the support. This initial shielding effect was substantially reduced after the catalytic tests in the PTA mode (Figure 9b). Taking into account both the catalytic and UV–Vis data, it can be supposed that PdO particles were initially located in the bulk of the support, and the high-temperature treatment facilitated their movement from the bulk to the surface. Additionally, a new band at 670 nm appeared along with the overall increase in the intensity of the charge transfer bands and the *d-d* transition bands. This behavior differs qualitatively from the behavior of Pd/Al_2_O_3_ catalysts at high temperatures (~1000 °C), which is accompanied by the formation of agglomerated Pd^0^ particles with a characteristic structureless band at wavelengths above 400 nm [57] and a decrease in the intensity of the PdO absorption bands. For the Pd/C12A7 and Pd@C12A7 catalysts, this phenomenon was not observed. Moreover, some signs of the further formation of the oxide structure were observable. This allowed for the conclusion that the Pd species predominated in the samples. Along with this, the formation of the CaPd_3_O_4_ species could be supposed. The similarity of the UV–Vis spectra of the Pd/C12A7 and Pd@C12A7 samples tested in the PTA mode correlated well with the catalytic results.

**Figure 10:**
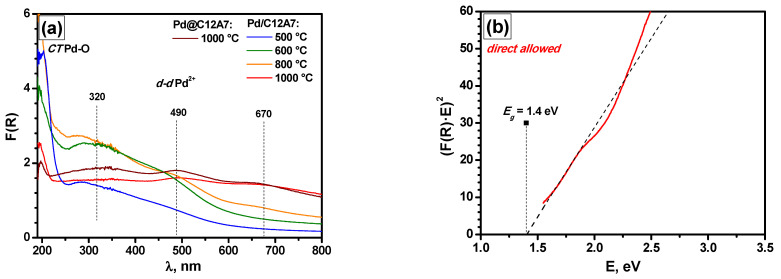
Diffuse reflectance UV–Vis spectra for the Pd/C12A7 sample consecutively calcined in the range of 500 to 1000 °C and the Pd@C12A7 sample after catalytic tests in the PTA mode (**a**). Dependencies of (*F(R)·E*)^2^ on photon energy *E* characteristic of band-gap (*E*_g_) values for direct allowed transitions for Pd/C12A7 after catalytic tests in the PTA mode (**b**).

In order to track the evolution of the samples in detail, they were calcined sequentially in cycles for 6 h, which was significantly longer than in the PTA experiments. The corresponding spectra are shown in Figure 10a. The beginning of the formation of the *d-d* band at 670 nm occurred at 800 °C, which corresponds to the conditions known for CaPd_3_O_4_ formation [56,58]. Moreover, the spectra of Pd/C12A7 and Pd@C12A7 after calcination at 1000 °C were also similar. The decrease in the intensity of the charge transfer bands in relation to the spectra after the PTA tests (Figure 9b) was presumably due to the sintering of the support and the appearance of CaPd_3_O_4_ species.

For the Pd/C12A7 sample after the PTA tests (Figure 10b), the characteristic *E*_g_ value for direct allowed transitions was ~1.4 eV. Compared to the typical *E*_g_ values of PdO (2.35–0.8 eV, depending on the particle size), the measured value seems to be quite reasonable. Taking into account the assumption made regarding the formation of CaPd_3_O_4_, which has an *E*_g_ of ~0.25 eV [56], additional measurements in a spectral region widened to ~5 μm are required.

## 3. Conclusions

In the present paper, palladium-containing catalysts based on aerogel-prepared mayenite were developed. Two preparation approaches were used: addition of palladium nitrate to the Ca(OH)_2_-Al(OH)_3_ gel and wet impregnation of the preliminarily prepared C12A7 support. The introduction of palladium slightly worsened the textural characteristics of the samples. Greater dispersion of PdO species was observed for Pd/C12A7 compared to Pd@C12A7. According to the EPR spectroscopy, the palladium-containing samples possessed fewer electron-donor and electron-acceptor sites than the original support. Nevertheless, the presence of reactive oxygen species capable of forming nitroxyl radicals in reaction with DPA was proven for both samples.

The TPR experiments in the hydrogen and CO atmospheres, along with catalytic tests, confirmed the inferior dispersity of Pd in the case of the Pd@C12A7 sample. The TPR tests also revealed the presence of reactive oxygen species in the samples and the possibility of regeneration of these species through oxidative treatment with air at 500 °C. In terms of catalytic performance, the Pd/C12A7 sample showed significantly higher activity and better stability. Even after being calcined at 1000 °C, it showed no significant loss of catalytic activity. The state of mayenite-supported Pd differs significantly from that of conventional Pd/Al_2_O_3_ catalysts. Thus, diffuse reflectance UV–Vis spectroscopy revealed the formation of CaPd_3_O_4_ for the mayenite-supported samples, which could explain the observed Pd stabilization effect. To summarize, the impregnation method made it possible to achieve higher dispersity for the Pd species in the catalyst based on aerogel-prepared mayenite. This was reflected in the higher activity of the Pd/C12A7 sample in catalytic CO oxidation and higher reactivity with regard to H_2_ and CO consumption.

## 4. Materials and Methods

### 4.1. Preparation of the Samples

The mayenite sample was prepared as described elsewhere [26,27,59]. In order to synthesize an aerogel with a composition of 12CaO-7Al_2_O_3_ (labeled C12A7), the sol-gel method involving a supercritical drying procedure was used. In the first step, 50 mL of methanol (J.T. Baker, Phillipsburg, NJ, USA) was added to 0.82 g of calcium metal (abcr GmbH, Karlsruhe, Germany). The mixture was refluxed for 1 h at 70 °C. Next, 4.28 g of aluminum isopropoxide (Alfa Aesar, Ward Hill, MA, USA), 40 mL of isopropanol (EKOS-1, Moscow, Russia), and 100 mL of toluene (Baza1r, Staraya Kupavna, Russia) were added to the formed calcium methoxide solution. In the following stage, hydrolysis was carried out with a stoichiometric amount of distilled water. The formed Ca(OH)_2_-Al(OH)_3_ gel was stirred overnight. Then, the gel was put in an autoclave and heated in an argon atmosphere for 3 h to a temperature of 265 °C. The pressure in the autoclave rose up to 80 atm before being slowly released at this temperature. After the autoclave drying procedure, the sample was calcined at 500 °C.

Palladium was introduced into the composition of the C12A7-based catalysts through two approaches. First, a calculated amount of a commercial palladium nitrate solution containing 14.89 wt% of palladium (Krastsvetmet, Krasnoyarsk, Russia) was mixed with 5 mL of ethanol and added dropwise to the freshly prepared Ca(OH)_2_-Al(OH)_3_ gel. The drying and calcination procedures were carried out similarly to that described above for C12A7. The loading of palladium was 1 wt% (with respect to the calcined sample). This sample was denoted Pd@C12A7. Second, an incipient-wetness impregnation method was used. Commercial palladium nitrate solution in a water–ethanol mixture was added evenly to the C12A7 sample. The impregnated support was calcined at 500 °C for 6 h. The loading of palladium in this sample was also 1 wt%. This sample was denoted Pd/C12A7.

### 4.2. Characterization of the Samples

The textural characteristics of the samples were determined with low-temperature nitrogen adsorption/desorption using an Autosorb-6B-Kr instrument (Quantum Instruments, Boynton Beach, FL, USA). Initially, the samples were degassed under a dynamic vacuum at 300 °C. Nitrogen adsorption/desorption isotherms were recorded at 77 K. The Brunauer–Emmett–Teller (BET) method was used to calculate specific surface area (SSA). The total specific pore volume *V*_p_ was calculated from the nitrogen volume adsorbed at a relative pressure p/p° = 0.991.

The structure and microstructure of the samples were studied with high-resolution transmission electron microscopy (HR TEM) using a JEM 2010 electron microscope (JEOL, Tokyo, Japan) with an acceleration voltage of 200 kV and a lattice limit resolution of 0.14 nm. The images were recorded using the CCD-matrix Soft Imaging System (Münster, Germany). The device is equipped with an XFlash energy-dispersion X-ray (EDX) spectrometer (Bruker AXS GmbH, Karlsruhe, Germany) with a semi-conductor Si-detector with an energy resolution of 130 eV. For electron microscopy studies, the samples were deposited on perforated carbon substrates fixed on a copper or molybdenum grid using an ultrasonic dispersant, which made it possible to achieve uniform particle distribution across the substrate surface.

The EPR spectra were registered under ambient conditions using an ERS-221 X-band EPR spectrometer (Center of Scientific Instruments Engineering, Leipzig, Germany). 1,3,5-Trinitrobenzene (TNB), phenothiazine (PTZ), and diphenylamine (DPA) probes were adsorbed from 2·10^−2^ M solutions in toluene. Prior to their adsorption, the samples were heated in air at 500 °C for 2 h. After activation, the samples were quickly cooled down to room temperature and filled with a spin probe solution. The EPR spectra were recorded at room temperature immediately after the adsorption of the probe and after the heating at 80 °C for 18 h. Such heating contributes to the polycondensation processes and to the formation of structures with a lower ionization potential [33]. This allows the identification of weak electron-acceptor and electron-donor sites. The concentrations of paramagnetic particles were calculated through double integration of spectra using a standard.

Diffuse reflectance UV–Vis spectra were recorded between 190 and 800 nm using a Varian Cary 300 UV/VIS Bio UV–Vis spectrometer (Agilent Technologies, Palo Alto, CA, USA) with a DRA-CA-3300 integrating sphere (Labsphere, North Sutton, NH, USA) and a Spectralon standard as a reference. The UV–Vis spectra were transformed into the Kubelka–Munk function *F(R)* = *α/s* [60], where *α* is the absorption and *s* is the scattering. All spectra of the supported samples are given with subtraction of the absorption spectrum of the support. The band gap (*E*_g_) was determined from the diffuse reflection spectra using an expression proposed by Tauc, Davis, and Mott:

[F(R)·hv]^n^ = A(hv−E_g_) (1)

where *h* is the Planck’s constant, *ν* is a frequency of vibration, function *F(R)~α* is an absorption coefficient, *E*_g_ is a band gap, and *A* is a coefficient. *hv* represents the energy per photon, *n* = 1/2 means an indirect allowed transition, and *n* = 2 represents a direct allowed transition [61].

The phase composition of the samples was determined through powder X-ray diffraction (XRD) analysis using a D8 Advance diffractometer (Bruker AXS GmbH, Karlsruhe, Germany) equipped with a CuK_α_ radiation source. The data were collected in a 2θ range of 10 to 75° with a step of 0.05° and a collection time of 3 s. The crystallite size of the samples was calculates using the Scherrer equation [62]:

D = Kλ/βcosθ(2)

where *D* is an average crystallite size, *K* is a constant (equal to 0.9), *λ* is a wavelength of the X-ray, *β* is a line broadening at FWHM, and *θ* is the Bragg’s angle.

The redox properties of the samples were studied by means of the temperature-programmed reduction (TPR) technique. The experiments were performed in an H_2_ (TPR-H_2_) or CO (TPR-CO) atmosphere. A specimen (200 mg; grain size of 0.25–0.5 mm) was loaded into a quartz reactor. The reactor was fed with a mixture consisting of 10 vol% H_2_ (TPR-H_2_) or 10 vol% CO (TPR-CO) and 90 vol% N_2_ at a flow rate of 57.8 mL/min and heated up to 700 °C with a ramping rate of 10 °C/min. The concentrations of H_2_ or CO and CO_2_ at the reactor outlet were measured using GAMMA-100 gas analyzers (FSUE SPA Analitpribor, Smolensk, Russia) equipped with a thermal conductivity detector (for H_2_) or an opto-acoustic detector (for CO and CO_2_). After the reduction stage, the reactor was cooled down to room temperature. Then, the re-oxidation stage was carried out in an air flow of 10 mL/min. The reactor was heated up to 500 °C with a ramping rate of 10 °C/min and held at this temperature for 1 h. For each sample, three redox cycles were performed in the hydrogen atmosphere, and then another three redox cycles were performed in the CO atmosphere.

A model reaction of CO oxidation was used to compare the catalytic activity of the samples. In order to access the thermal stability, a prompt thermal aging (PTA) mode was used. A specimen (300 mg; grain size of 0.25–0.5 mm) was loaded into a quartz reactor. The reactor was fed with a reaction mixture containing CO (0.15 vol%), propane (36 ppm), propene (50 ppm), toluene (14 ppm), NO (0.015 vol%), oxygen (14 vol%), and nitrogen (balance). Each sample was subjected to 11 catalytic cycles. During these cycles, the reactor was heated from 50 °C up to the final temperature, which was 320 °C (for the first and second cycles), 600 °C (for the third and fourth cycles), 800 °C (for the fifth and sixth cycles), 900 °C (for the seventh and eighth cycles), 1000 °C (for the ninth and tenth cycles), and 500 °C (for the eleventh cycle). The ramping rate was 10 °C/min. The flow rate of the reaction mixture was 334 mL/min. At the reactor outlet, the reaction mixture was analyzed with an ULTRAMAT 6 gas analyzer (Siemens, Munich, Germany) equipped with an infrared gas sensor. The CO concentration was registered every 2 s. The temperatures of 50% and 90% CO conversion (T_50_ and T_90_) were used as the criteria of the catalytic activity.

## Figures and Tables

**Table 1:** Textural characteristics of pure and Pd-containing C12A7 samples.

Sample	Calcination Temperature, °C	SSA, m^2^/g	V_pore_, cm^3^/g
C12A7	As-prepared	327	1.41
	500	247	0.95
	700	147	0.61
	900	18	0.06
Pd/C12A7	500	200	1.0
Pd@C12A7		180	1.1

**Table 2:** Concentrations of radical species (10^18^ g^−1^) observed after the interaction of spin probe molecules to the active sites on the surfaces of C12A7, Pd/C12A7, and Pd@C12A7 samples.

Sample	Diphenylamine		Phenothiazine		1,3,5-Trinitrobenzene	
	After Activation at 500 °C	After Heating at 80 °C for 18 h	After Activation at 500 °C	After Heating at 80 °C for 18 h	After Activation at 500 °C	After Heating at 80 °C for 18 h
C12A7	2.45	-	3.55	33	0.83	8.13
Pd/C12A7	1.06	-	0.85	1.58	0.54	0.76
Pd@C12A7	1.48	-	1.34	1.44	0.44	0.46

**Table 3:** Quantitative data for TPR-H_2_ and TPR-CO experiments.

Sample	Cycle Number	TPR-H_2_		TPR-CO		
		H_2_ Uptake, µmol/g		CO Uptake, µmol/g		CO_2_ Released, µmol/g
		<200 °C	>200 °C	<200 °C	>200 °C	
Pd/C12A7	1	71	3539	23	1322	666
	2	83	423	51	1521	1103
	3	71	433	37	1509	942
Pd@C12A7	1	79	2624	22	1024	924
	2	81	209	24	1344	1476
	3	32	152	42	1501	1315

**Table 4:** Comparison of the catalytic performance data (T_50_) for the studied samples and recently reported ones.

Sample	Cycle Number							Ref.
	1	2	3	4	5	6	7	
Pd/C12A7	208	214	215	250	253	265	270	This work
Pd@C12A7	225	231	236	264	266	281	285	This work
Pd_0.6_(Ir_0.2_Rh_0.8_)/Al_2_O_3_	219	256	260	271	272	277	278	[45]
Pt/Al_2_O_3_	286	281	279	278	275	265	262	[46]
Rh_0.4_Pt_0.6_/Al_2_O_3_	278	276	274	274	273	262	261	[46]
Rh_0.4_(Pd_0.75_Pt_0.25_)_0.6_/Al_2_O_3_	208	240	243	256	258	266	265	[46]

## Data Availability

Data are contained within the article.
